# Use of Urea Wash ELISA to Distinguish Zika and Dengue Virus Infections 

**DOI:** 10.3201/eid2407.171170

**Published:** 2018-07

**Authors:** Wen-Yang Tsai, Han Ha Youn, Jasmine Tyson, Carlos Brites, Jih-Jin Tsai, Celia Pedroso, Jan Felix Drexler, Angel Balmaseda, Eva Harris, Wei-Kung Wang

**Affiliations:** University of Hawaii at Manoa, Honolulu, Hawaii, USA (W.-Y. Tsai, H.H. Youn, J. Tyson, W.-K. Wang);; Federal University of Bahia, Salvador, Brazil (C. Brites, C. Pedroso);; Kaohsiung Medical University, Kaohsiung, Taiwan (J.-J. Tsai);; University of Bonn Medical Centre, Bonn, Germany (J.F. Drexler);; National Center for Diagnosis and Reference, Ministry of Health, Managua, Nicaragua (A. Balmaseda);; University of California at Berkeley, Berkeley, California, USA (E. Harris)

**Keywords:** Zika virus, dengue virus, nonstructural protein 1, serologic test, urea, viruses

## Abstract

Serologic testing remains crucial for Zika virus diagnosis. We found that urea wash in a Zika virus nonstructural protein 1 IgG ELISA distinguishes secondary dengue virus infection from Zika virus infection with previous dengue (sensitivity 87.5%, specificity 93.8%). This test will aid serodiagnosis, serosurveillance, and monitoring of Zika complications in dengue-endemic regions.

The rapid spread of Zika virus and its association with fetal microcephaly and other birth defects (congenital Zika syndrome) present a pressing need for sensitive and specific diagnostic tests (*1*,*2*). Centers for Disease Control and Prevention guidelines for laboratory diagnosis of Zika virus infection include a positive reverse transcription PCR as soon as possible after symptom onset to confirm Zika virus and a negative IgM test result to exclude Zika virus (*3*). Serologic testing remains a crucial component of Zika diagnosis because most Zika virus infections are asymptomatic, many persons seek Zika virus testing beyond the period during which RNA is detectable, and Zika virus can be transmitted sexually or after asymptomatic infection (*1*–*3*).

Zika virus belongs to the family *Flaviviridae*, in which several arboviruses, including the 4 serotypes of dengue virus (DENV-1–4), cause substantial disease in humans. Because of cross-reactivity of antienvelope antibody to Zika virus and other flaviviruses, positive or equivocal IgM results based on envelope protein require further testing with plaque-reduction neutralization tests (*3*–*5*). These tests can confirm acquisition of Zika virus as the first flavivirus infection (primary Zika virus [pZIKV] infection) but are more challenging to interpret for those who have experienced previous flavivirus infections.

Several studies have demonstrated that DENV–immune serum and monoclonal antibodies can enhance Zika virus replication in vitro and in vivo (*6*–*9*) and raised concerns that previous DENV infection might increase the risk for and severity of congenital Zika syndrome. A recent study reported that a nonstructural protein 1 (NS1)–based blockade of binding ELISA can distinguish Zika virus and other flavivirus infections (*10*). However, it cannot distinguish pZIKV, Zika virus infection with previous dengue (DENV-ZIKV), and secondary DENV (sDENV) infections, which is critical in Zika virus– and DENV-endemic regions.

## The Study

The Institutional Review Board of the University of Hawaii approved this study of coded serum or plasma samples (CHS #17568, CHS #23786). Convalescent-phase samples from patients with confirmed Zika virus infection who were either DENV-naive (designated as pZIKV panel) or previously exposed to DENV (designated as DENV-ZIKV panel) were from a cohort study in Nicaragua (*11*) (Table). Convalescent-phase samples from patients who had symptoms compatible with Zika virus infection and detectable anti-DENV IgG during the acute phase (probable DENV-ZIKV panel) came from Bahia, Brazil (*12*). Convalescent-phase or post–convalescent-phase (3 months–6 years after symptom onset) samples from patients who had confirmed primary DENV (pDENV) or sDENV infection came from Taiwan, Hawaii (USA), and Nicaragua; 12 flavivirus-naive samples had been previously described (*12*,*13*).

**Table:** Sampling time, serotype, and sources of serum/plasma panels in study of use of urea wash ELISA to distinguish Zika and dengue virus infections*

Panel sample collection times	Category	Sampling time after symptom onset, mean (range)	No. patients	Source (no. patients) and year(s) of sample collection	Shown in
Single time point					
pDENV-1	Convalescent to postconvalescent	138 (19−263) d	16	Taiwan (4), 2001–2002; Hawaii, USA (12), 2015	Figure 1
pZIKV	Convalescent	17 (14−24) d	20	Nicaragua, 2016	Figure 1
sDENV	Convalescent	14 (8−35) d	24	Taiwan, 2001–2002	Figure 1
DENV-ZIKV	Convalescent	16 (14−19) d	20	Nicaragua, 2016	Figure 1
Probable DENV-ZIKV	Convalescent	10 (6−14) d	19	Brazil, 2015–2016	Figure 1
sDENV	Postconvalescent	3.2 (3−4) mo	6	Taiwan (2), 2006–2009; Nicaragua (4), 2006–2008	Figure 2
sDENV	Postconvalescent	12 (12−12) mo	18	Nicaragua, 2006–2008	Figure 2
sDENV	Postconvalescent	19.7 (18−24) mo	14	Taiwan (10), 2006–2009; Nicaragua (4), 2006–8	Figure 2
sDENV	Postconvalescent	71 (67−72) mo	5	Taiwan, 2006–2009	Figure 2
Sequential time points					
sDENV	Postconvalescent	10 (3−18) mo	3	Nicaragua, 2006–2008	Figure 2

*DENV-ZIKV, ZIKV infection with previous dengue; pDENV-1, primary dengue virus 1 infection; pZIKV, primary Zika virus infection; sDENV, secondary dengue virus infection. †3–4 samples/patient.

The expression and purification of Zika virus NS1 protein (strain HPF2013) have been described (*12*). Purified DENV-1 NS1 protein was from the Native Antigen Company (Oxford, UK). NS1-IgG and NS1-IgM ELISAs as well as cutoff, positive, and negative controls in each plate have been described (*12*). The relative optical density (rOD) values were OD divided by the mean OD of positive controls. For the urea wash, we added 100 μL urea (4–8 mol/L) to each well at room temperature for 5 min between the second and third washings of NS1-IgG ELISA after the primary antibody (total 4 washings) (*14*). We used the 2-tailed Mann-Whitney test to determine p values comparing 2 groups (GraphPad Prism 6, https://www.graphpad.com/scientific-software/prism). 

**Figure 1:**
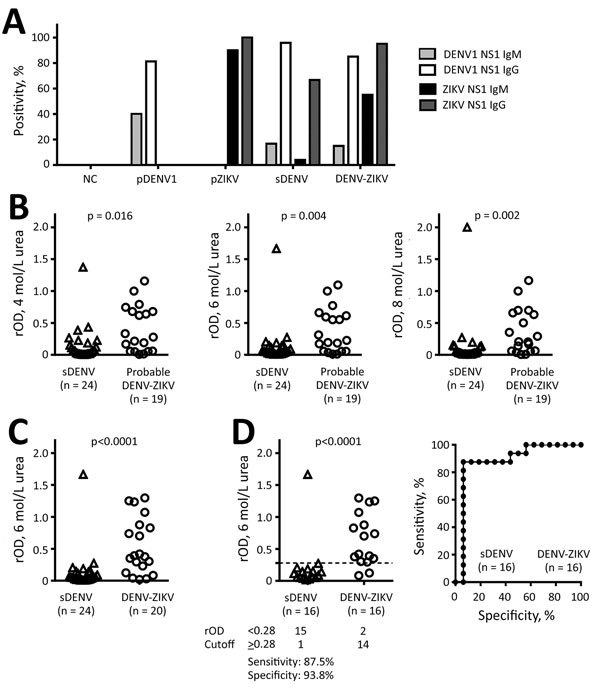
NS1 IgM and IgG ELISAs and urea wash in ZIKV–NS1 IgG ELISA. A) Positivity rates for each panel. Only samples collected <3 months after symptom onset were tested for IgM. B) sDENV infection and probable DENV-ZIKV panels were tested with different concentrations (4, 6, and 8 mol/L) of urea wash. C, D) sDENV and DENV-ZIKV panels were tested with 6 mol/L urea wash: C) all samples; D) samples positive for both DENV-1–NS1 and ZIKV-NS1 IgG ELISAs. Sensitivity and specificity are based on relative optical density cutoff at 0.28 (dashed line). Receiver-operating characteristics are shown in the graph on the right. Data are the mean of 2 experiments (each in duplicate). The 2-tailed Mann-Whitney test was used. DENV, dengue virus; DENV-ZIKV, confirmed Zika virus infection with previous exposure to DENV; NS1, nonstructural protein 1; pDENV1, primary DENV-1 infection; pZIKV, primary ZIKV infection; rOD, relative optical density; sDENV, secondary DENV infection; ZIKV, Zika virus.

To evaluate convalescent-phase samples from pDENV1, pZIKV, sDENV, and DENV-ZIKV panels, we used 4 ELISAs. The primary DENV1 and pZIKV panels recognized their own NS1 without cross-reactivity (Figure 1, panel A; Technical Appendix Table 1). The DENV-ZIKV panel recognized Zika virus and DENV NS1. The sDENV panel recognized not only DENV but also Zika virus NS1, especially in IgG ELISA, suggesting that cross-reactivity in NS1 IgG ELISA between sDENV and DENV-ZIKV panels is a challenge for NS1-based serologic tests for Zika virus infection.

We next investigated whether a urea wash in Zika virus NS1 IgG ELISA could distinguish sDENV and DENV-ZIKV infections. Different concentrations (4, 6, and 8 mol/L) of urea wash resulted in significantly lower rODs in the sDENV panel than in the probable DENV-ZIKV and DENV-ZIKV panels (Figure 1, panels B, C). We chose the 6 mol/L urea wash for further analysis, considering its optimal cutoff value (data not shown). Comparing the samples with positive Zika virus– and DENV-1–NS1 IgG ELISA results (Figure 1, panel D), a cutoff rOD of 0.28 can distinguish the 2 panels with 87.5% sensitivity and 93.8% specificity.

**Figure 2:**
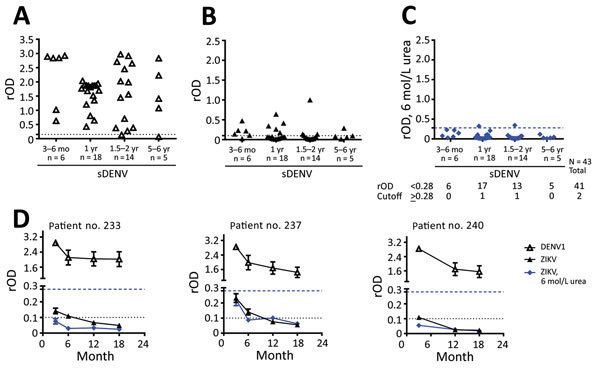
NS1 IgG ELISAs with urea wash for sDENV infection panel over time. A) DENV-1–NS1 IgG ELISA; B) ZIKV–NS1 IgG ELISA; and C) ZIKV-NS1 IgG ELISA with 6 mol/L urea wash for sDENV samples collected from 3 months to 6 years after symptom onset. D) Sequentially collected samples from 3 patients with sDENV infection. Dotted lines indicate relative optical density cutoffs of ELISAs; dashed lines indicate rOD cutoff (0.28) of ELISA with 6 mol/L urea wash. Data are expressed as mean ± SD (for panel D) of 2 experiments (each in duplicate). The 2-tailed Mann-Whitney test was used. DENV, dengue virus; NS1, nonstructural protein 1; rOD, relative optical density; sDENV, secondary DENV infection; ZIKV, Zika virus.

We further investigated whether a 6 mol/L urea wash could reduce IgG cross-reactivity to ZIKV-NS1 by sDENV panel at later times. For the 43 post–convalescent-phase samples, positivity rates in DENV-1–NS1 IgG ELISAs decreased from 100% (3–6 months after symptom onset) to 80% (5–6 years) and for ZIKV-NS1 IgG ELISAs from 83.3% to 40%, respectively (Figure 2, panels A, B). After 6 mol/L urea wash in ZIKV-NS1 IgG ELISA, rOD decreased greatly, resulting in 4.7% having an rOD >0.28 (Figure 2, panel C). Results for sequential samples from 3 patients with sDENV infection (Figure 2, panel C) were generally consistent with the results from cross-sectional samples; rODs were all <0.28 after 6 mol/L urea wash (Figure 2, panel D).

Although neutralization tests can confirm pZIKV infection, they remain difficult to interpret for patients who have previously experienced flavivirus infections, including sDENV and DENV-ZIKV infections. A recent study reported reduced cross-neutralization against Zika virus among samples from patients with sDENV infection >6 months after symptom onset; however, 23% still cross-neutralized Zika virus (*15*). Our findings suggest that a 6 mol/L urea wash in ZIKV-NS1 IgG ELISA can distinguish DENV-ZIKV and sDENV panels. It is conceivable that during sDENV infection, memory B cells recognizing NS1 residues that are conserved within the DENV serocomplex and between DENV and Zika virus expand greatly and generate high-avidity anti-NS1 antibodies through affinity maturation (*9*,*13*). During Zika virus infection among those with previous DENV infection, memory B cells recognizing NS1 residues conserved between DENV and Zika virus will expand and generate high-avidity antibodies. Moreover, naive B cells recognizing Zika virus–specific NS1 residues will also expand; the combination of these 2 types of anti-NS1 antibodies may contribute to anti-NS1 antibodies with higher avidity, which remain bound after urea wash, compared with those from the sDENV panel (Figure 1, panel C; Technical Appendix Figure 1).

This study has limitations. First, we tested only convalescent- and post–convalescent-phase samples. Second, the sample size in each panel was small; future studies with larger samples, including acute-phase and more sequential samples, are needed to validate these observations. Third, because our previous study showed cross-reactivity of anti-DENV NS1 antibodies within the DENV serocomplex (*5*), we chose only DENV-1-NS1 IgG ELISA for this study; there was no difference in the positivity rates of DENV-1-NS1 IgG ELISA between primary DENV-1 and sDENV-2 panels and between sDENV-1, sDENV-2, and sDENV-3 panels (Technical Appendix Table 2). Fourth, given the global spread of Zika virus to regions where different flaviviruses are prevalent, development of serodiagnostic assays to distinguish Zika virus and other medically relevant flaviviruses remains to be explored.

## Conclusions

Our method of combined ELISAs plus 6 mol/L urea wash in Zika virus-NS1 IgG ELISA is simple, cost-effective, and applicable for use at field sites. This method could be used for routine serologic testing for Zika virus in dengue-endemic regions and for serosurveillance and Zika pregnancy studies to clarify epidemiology, transmission, and complications (*1*–*3*). Because congenital Zika syndrome may affect infants during growth and development, IgG-based NS1 ELISAs plus 6 mol/L urea wash could be used in retrospective studies to elucidate the contribution of pZIKV infection alone or Zika virus infection with previous DENV to the full spectrum of congenital Zika syndrome (*1*,*2*).

Technical Appendix Supplemental results for study of using urea wash ELISA to distinguish Zika and dengue virus infections.
