# Supplementary material for: Use of Urea Wash ELISA to Distinguish Zika and Dengue Virus Infections
Source: Emerg Infect Dis. 2018 Jul;24(7):1355–9. doi: 10.3201/eid2407.171170 (PMC6038735; doi:10.3201/eid2407.171170)
Supplement: Technical Appendix — Supplemental results for study of using urea wash ELISA to distinguish Zika and dengue virus infections. [file 17-1170-Techapp-s1.pdf]

Article DOI: https://doi.org/10.3201/eid2407.171170

Use of Urea Wash ELISA to Distinguish Zika and Dengue Virus Infections

Technical Appendix

Technical Appendix Table 1. Results of ELISA in different serum/plasma panels*

	serum/plasma panels				
	Naïve	pDENV1	pZIKV	sDENV	Dengue-ZIKV
ELISA	n = 12	n = 16	n = 20	n = 24	n = 20
DENV1-NS1 IgM+	0/12 (0%)	2/2 (100%)†, 2/5 (40%)	0/20 (0%)	4/24 (16.7%)	3/20 (15%)
DENV1-NS1 IgG+	0/12 (0%)	13/16 (81.3%)	0/20 (0%)	23/24 (95.8%)	17/20 (85%)
ZIKV-NS1 IgM+	0/8 (0%)	0/5 (0%)	18/20 (90%)	1/24 (4%)	11/20 (55%)
ZIKV-NS1 IgG+	0/12 (0%)	0/16 (0%)	1/20 (5%)†, 18/18 (100%)	16/24 (66.7%)	19/20 (95%)

*Results were based on data from two experiments (each in duplicates). pDENV1, primary DENV1 infection; pZIKV, primary ZIKV infection; sDENV: secondary DENV infection; dengue-ZIKV, ZIKV infection with previous dengue; ELISA, enzyme-linked immunosorbent assay; PSO: post-symptom onset.
†Only samples collected <3 mo PSO were tested for IgM. IgM+ rate equals 2/2 (100%) and 2/5 (40%) for samples collected <1 mo and <3 mo PSO, respectively.
‡IgG+ rate equals 1/20 (5%) and 18/18 (100%) for convalescent-phase and follow-up samples, respectively.

Technical Appendix Table 2. Results of DENV1-NS1 IgG ELISAs in samples from patients with different DENV infections*

Samples from patients with different DENV infections	Sampling time PSO	Number patients	DENV1-NS1 IgG ELISA+	Presented in
pDENV1	Convalescent to post-convalescent (19 d–9 mo)	16	13/16 (81.3%)	Figure 1
sDENV2	Convalescent (8–35 d)	24	23/24 (95.8%)	Figures 1
sDENV2	Post-convalescent (3–72 mo)	27	25/27 (92.6%)	Figure 2
sDENV1	Post-convalescent (3–24 mo)	5	5/5 (100%)	Figure 2
sDENV3	Post-convalescent (12–72 mo)	11	11/11 (100%)	Figure 2

*PSO, post-symptom onset; pDENV1, primary DENV1 infection; sDENV1, sDENV2 and sDENV3: secondary DENV1, DENV2 and DENV3 infections, respectively. p = 0.28, 1, 1 and 1, comparing pDENV1 (n = 16) vs. sDENV2 (n = 24), sDENV2 (n = 27) vs. sDENV1 (n = 5), sDENV2 (n = 27) vs sDENV3 (n = 11), and sDENV1 (n = 5) vs. sDENV3 (n = 11), respectively, Fisher exact test.

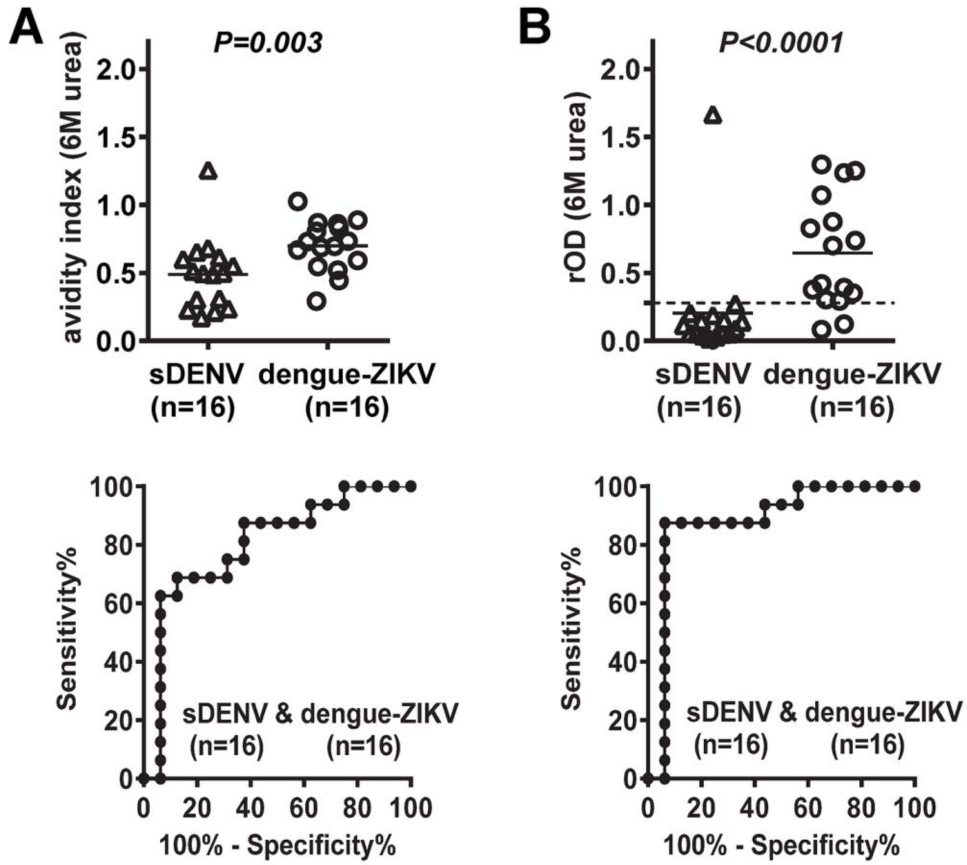

Technical Appendix Figure. Receiver-operating characteristics (ROC) analysis of avidity index and rOD values. The ZIKV-NS1 IgG ELISA with 6 M urea wash was tested for samples from sDENV and dengue-ZIKV panels, which were positive for both ZIKV- and DENV1-NS1 IgG. (A) avidity index and its ROC analysis. avidity index = rOD with 6 M urea/rOD without 6 M urea. (B) rOD values and its ROC analysis. Dashed lines indicate cutoff rOD value (at 0.28) with 6 M urea wash. Data are the mean of two experiments (each in duplicate). The two-tailed Mann-Whitney test was used.
